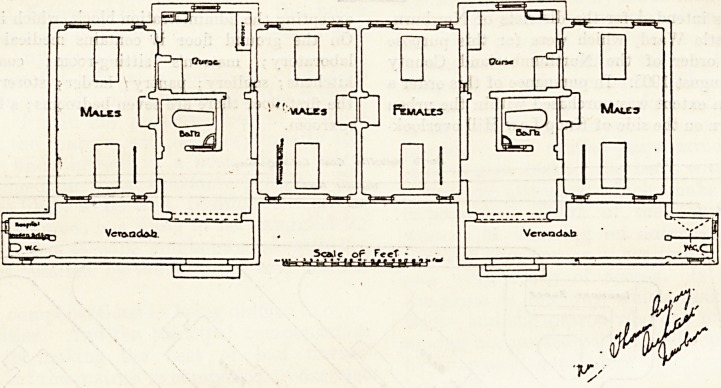# Hospital for Infectious Disease, Newburn, Northumberland

**Published:** 1905-10-14

**Authors:** 


### HOSPITAL FOR INFECTIOUS DISEASE, NEWBURN, NORTHUMBERLAND.

This Hospital is intended for the districts of Newburn, Gosforth, and Castle Ward, which were for this purpose united under an order of the Northumberland County Council made in August 1903. In pursuance of this order a site of two acres in extent was purchased within the urban district of Newburn on the side of Knop Law Hill overlooking the Tyne Valley. A sum of £9,500 was borrowed, and of this £7,964 was set aside for constructing the building and laying out the grounds.

The hospital consists of five blocks: namely, the administration, the laundry, the observation, the scarlet fever, and the typhoid block. These blocks are of one story only excepting the administration block, which is of two stories. On the ground floor it contains medical officers' room; laboratory; matron's sitting-room; committee room; kitchens; scullery; pantry; larder; storerooms, etc. On the first floor there are seven bedrooms; a bathroom, and a boxroom.

The laundry is well arranged and compact; but there is nothing special about it. Attached to this block are an ambulance house and a mortuary.

The plot of land on which the blocks stand is a parallelogram, running nearly north and south. The north boundary abuts on the road leading from Newburn to Newcastle.

The patients' blocks being placed diagonally face north-east and south-west. This is scarcely an ideal aspect for a hospital; but as no individual patient is likely to be more than a few weeks in the wards it is not very objectionable.

The observation block has one front facing the laundry and its west end facing the administration block. The block is divided into two equal sections. The west end has a double-bedded room for males, and next to this is a nurses' room and a bathroom—then another two-bedded ward for males. Further east is a two-bedded room for females; then another nurses' room, and at the extreme east end is a two-bedded ward for males. This arrangement seems at first glance a little peculiar, and it is not evident to us why all the male wards were not placed together; but of course this is rather a matter of naming than of planning. Verandahs are placed along the south-west fronts, and at the ends of these verandahs are the w.c.'s. All the wards in this block have good cross-ventilation, but the end wards would have been more cheerful if each had had at least one window in the long wall.

The scarlet fever pavilion consists of a centre and two wings. The centre, beginning at the south-west front, has a good-sized verandah from which opens the vestibule leading to a central hall having the bathroom on the right hand side, and the nurses' duty room on the north-east front. This room is correctly fitted up and has inspection windows looking into the wards. These wards are approached from the central hall, and each contains six beds; those in the north-east wing being for men and those in the south-west wing being for women. Each bed has a window on both sides; and the cubic space per bed seems ample; but here again we should like to have seen windows in the free ends of the wards. The sanitary annexes are properly placed and efficiently cut off from the wards by cross-ventilated passages.

The typhoid pavilion is a facsimile of the scarlet fever one.

The wards in the observation pavilion are heated by Shorland's grates; and fresh air is supplied by low-level ventilators and by inlets placed five feet above the floor line; while the foul air is drawn off by Boyle's extractors; and all the ward windows are arranged with hopper fanlights opening inwards. The fever blocks are heated by double-fronted stoves with ascending flues, having an outer casing which delivers warm air into the room. The casings are carried above the level of the ceilings to form extractors, and are connected by zinc tubing to ventilators fixed in the ward ceilings. One would like to ask how these flues and tubes are to be kept free from dust.

The drainage is good. Each block being separately drained, intercepted, and ventilated. The water is supplied by the Newcastle and Gateshead Water Company; and the electric light by the District Supply Company. Gas is also laid on.

The architect was Mr. Thomas Gregory of Newburn-on-Tyne, and the contractors were Messrs. Davison and Bolam.

## Figures and Tables

**Figure f1:**
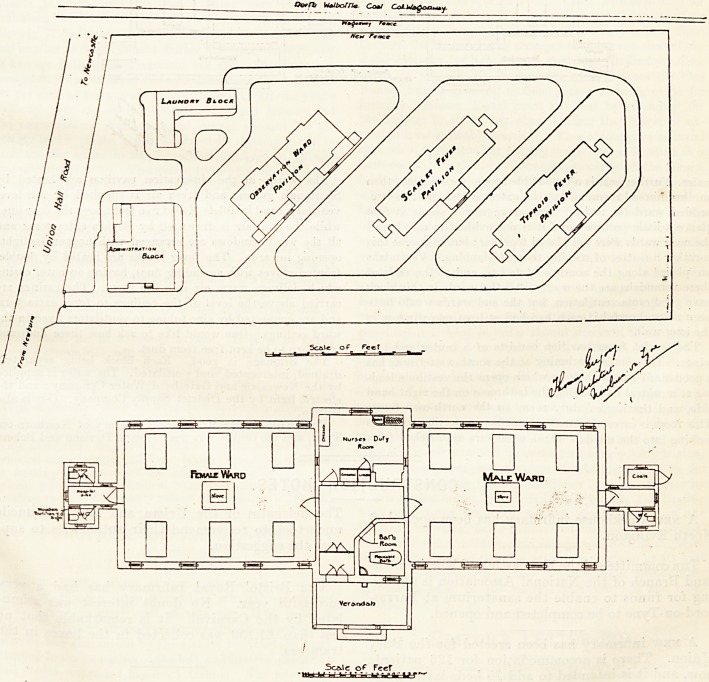


**Figure f2:**